# Psychometric evaluation of the Parental Reflective Functioning Questionnaire in Polish mothers

**DOI:** 10.1371/journal.pone.0299427

**Published:** 2024-04-17

**Authors:** Anna Kamza, Patrick Luyten, Konrad Piotrowski

**Affiliations:** 1 Center for Research on Personality Development, Institute of Psychology, SWPS University, Poznań, Poland; 2 Faculty of Psychology and Educational Sciences, KU Leuven, Leuven, Belgium; 3 Research Department of Clinical, Education and Health Psychology, UCL, London, United Kingdom; Qatar University College of Nursing, QATAR

## Abstract

Parental reflective functioning (PRF) refers to a parent’s capacity to reflect on and understand the inner mental states of their child, their own mental states with regard to their child, and how these mental states may influence their behavior and interactions. This capacity has been shown to foster secure attachment in children and their socio-emotional development. The present study examined the psychometric properties of the Polish translation of the Parental Reflective Functioning Questionnaire (PRFQ), a brief screening measure of PRF, in a large community sample of Polish mothers of children aged 0–5 years (*N* = 979). Confirmatory factor analysis supported the hypothesized three-factor structure of the PRFQ, which consists of three subscales: prementalizing modes, certainty about mental states, and interest and curiosity in mental states. However, item loadings suggested that the 15-item version fitted the data better than the original 18-item version. These three PRFQ subscales exhibited satisfactory and moderate six-month test–retest reliability. They also correlated in theoretically expected ways with several criterion measures such as maternal attachment, maternal parenting stress, parental role restriction, depression severity, and borderline symptoms. In conclusion, this study is the first to provide preliminary evidence for the reliability and validity of the PRFQ as a measure of parental reflective functioning in Polish mothers.

## Introduction

Parental reflective functioning (PRF), also referred to as parental mentalizing, represents the parent’s ability to recognize and understand the child’s mental states, such as thoughts, feelings, and intentions. It involves being attuned to the child’s perspective and recognizing that the child has a separate inner world [1–3]. PRF is considered an important feature of positive parenting, linked to responsive and sensitive behaviors and enhanced parent–child communication [4–6]. Parents with high levels of PRF are more likely to consider multiple factors influencing their children’s behavior and reflect on their own responses to it [7]. These parents tend to manage their emotional reactions more effectively, enabling them to better understand and resonate with their child’s emotions and respond compared to parents with lower levels of PRF [3]. Furthermore, reflective parents tend to engage more in meaningful and open dialogues with their children, helping them express their thoughts and emotions [8] and thus promoting the development of mentalizing in their offspring. However, there is evidence that mothers with low RF experience difficulties in managing their infants’ distress and exhibit heightened reactivity and overcontrolling behaviors [9]. This, in turn, leads to a low sense of attachment security in their children [9, 10]. The latter predicts difficulties in mentalizing and emotion regulation in children and is related to distortions in their psychosocial functioning [11].

Studies suggest that parents’ mentalizing is partly shaped by one’s attachment history and experiences of being mentalized by their caregivers in particular [4–6]. Parents with secure attachment in childhood tend to have a positive attachment model, which allows them to be more emotionally available and attuned to their child’s emotional needs. On the other hand, parents with insecure attachment might be preoccupied with attachment-related fears and insecurities, making it difficult to fulfill their parental roles reflectively. Insecure attachment and unresolved trauma are thought to lead to distortions in mentalizing, especially in an attachment-related context [12]. These two factors are often common in borderline personality disorder (BPD) [13–15]. Mothers with BPD symptoms struggle with, among others, instability in self-image, emotion regulation, and impulsivity, which makes it difficult for them to attune to their child’s inner world [14]. Fisher-Kern et al. [15] also noted that depressed mood, an important feature of BPD, itself leads to increases in arousal and stress. This impairs higher cognitive processes and thus is the basis for switching to prementalizing modes of thinking. Difficulties in mentalizing can lead to childrearing stress, characterized by perceptions of childrearing as burdensome and perceptions of parenthood as limiting and preventing from the lifestyle a parent would like to have (parental role restrictions) [16]. However, the links between PRF and stress are bidirectional, as heightened stress-related arousal inhibits the engagement of brain areas involved in mentalizing and emotion regulation [17].

### Assessment of parental reflective functioning

PRF is often scored on interviews and narratives such as the Parental Development Interview (PDI), the Working Model of Child Interview (WMCI), and the Adult Attachment Interview (AAI) [12, 18]. These methods provide a rich and nuanced understanding of a parent’s reflective capacities and their impact on the parent–child relationship. Luyten et al. developed the Parental Reflective Functioning Questionnaire (PRFQ) [1]. This brief screening tool is intended to be useful in clinical and nonclinical contexts, in studies with large sample sizes, and within several socioeconomic and educational backgrounds. The PRFQ is an 18-item multidimensional self-report measure primarily designed for parents of children aged 0–5 years. It includes three subscales that capture the critical facets of PRF, such as prementalizing modes, certainty about mental states, and interest and curiosity about mental states. *Prementalizing mode* (PM) addresses parents’ challenges in accurately perceiving their child’s mental state and interpreting the child’s behavior without considering the underlying mental processes [1, 14]. PM is expressed in parents’ tendency to make inaccurate and hostile attributions toward their children. High PM suggests significant distortions in PRF, which are commonly associated with certain forms of psychopathology such as, e.g., borderline personality disorder (BPD) or depression [13]. *Certainty about mental states* (CMS) portrays the extent to which parents acknowledge that mental states can be opaque and believe they comprehend their children’s inner world. Genuine mentalizing requires parents to be humble in their certainty about comprehending their child’s mental states. Deviations from the optimal level of CMS result in impaired mentalizing ability. Overconfidence in understanding mental states indicates hypermentalizing. Parents prone to hypermentalizing often overinterpret their children’s mental states and believe that they possess especially adept mentalizing abilities [1, 2, 12]. Consequently, they may arrive at hasty and misguided conclusions about their children, such as misjudging their intentions as malicious. Conversely, a low level of CMS, known as hypomentalizing, is linked to reduced confidence in making inferences about the inner world and limited overall engagement in mentalizing [12]. Parents with hypomentalizing may find it difficult to understand complex mental states and tend to rely on simplistic explanations for their children’s behavior. *Interest and curiosity about child’s mental states* (IC), finally, is an index of the parent’s interest in the child’s inner world. A very high IC may suggest intrusive hypermentalizing, whereas a very low IC may indicate hypomentalizing [1, 5, 12].

Numerous studies have replicated the three-dimensional structure of the PRFQ (e.g., [12, 19–24]) and have found adequate to good internal consistency for all subscales [1]. The convergent validity of the PRFQ was confirmed by its significant correlations with narrative measures of general reflective functioning [22, 25, 26] and task-based measures of mentalizing [27]. In addition, the three PRFQ dimensions are typically related in theoretically expected ways to criterion variables such as parental attachment dimensions, emotional availability, parenting stress, and infant attachment status [1, 10, 21, 23, 25, 30]. Yet, several studies found that some items of the original PRFQ showed low loadings on their hypothesized factor. The PRFQ has been translated and validated in a number of countries [19–24, 28–30]. However, unlike the original PRFQ, some adaptations (i.e., Canadian [19], Hungarian [22], Chinese [23], Italian [29], and American [30]) include fewer items because of low loadings of some of them in CFAs (this applies in particular to items 11 and 18). This suggests the possibility that there might be some semantic nuances in those items in different languages or cultures.

This study is the first to investigate the psychometric properties of the Polish version of the Parental Reflective Functioning Questionnaire [1] and its associations with maternal attachment dimensions, childrearing stress, perceived role restrictions resulting from being a mother, depressive symptoms, and borderline symptoms. Specifically, we tested the following hypotheses:

1. CFA will support the three-factor structure of the Polish version of the PRFQ in mothers, closely aligned with the theoretical descriptions of the critical features of PRF, as in the original PRFQ.
2. The correlations between the PRFQ subscales will be small to moderate, supporting a relative distinction between these aspects of parental reflective functioning. Based on previous studies, we expected the PM subscale to correlate negatively with the IC and CMS subscales and CMS and IC to correlate positively.
3. PM will be positively associated with indices of maladaptive maternal psychosocial functioning, i.e., maternal attachment anxiety and attachment avoidance, childrearing stress, role restrictions, depressive symptoms, and borderline symptoms; we expected the opposite pattern of associations for the CMS and IC subscales.

Given that very high and very low scores on the CMS and/or IC subscales have been suggested to be maladaptive [1, 15], we explored the possibility of a non-linear, U-shaped relationship between CMS/IC and our criterion variables. For example, very high scores on CMS and IC may reflect hypermentalizing, whereas very low scores on these subscales may reflect hypomentalizing.

## Materials and methods

### Design and participants

The data presented in this paper are part of a larger longitudinal, web -based study of parental difficulties in mothers of children aged 0–5 years. We used data from T1 (December 2021) and the 6-month follow up at T2 (June 2022) to assess the reliability of the PRFQ using a test–retest approach. Although the PFR may vary according to different factors (e.g., arousal, relationship-specific dynamics, or child developmental stage [5]), we expected PRFQ scores to remain at least moderately stable over this time interval.

We calculated the minimal sample size using Kim’s [31] formula for noncentrality parameter calculation for structural equation modeling (SEM) with a dedicated web-based calculator [32]. A minimal sample of 217 participants was required for CFA analysis with 18 items, three factors, *df* = 132, RMSEA of .05, power (1–β) of 95%, *p* < .05, and expected drop-out rate of 10%.

At T1, a total of 988 valid questionnaires were collected. However, after data inspection (see *Data analyses*section), the final sample consisted of 979 participants. They were biological mothers and all lived with their children. The frequency distribution of mothers based on children’s age was as follows: children below one year of age, 12%; 1-year-olds, 14%; 2-year-olds; 21% 3-year-olds; 24% 4-year-olds; 26% 5-year-olds. The remaining sociodemographic characteristics of the sample are shown in Table 1.

**Table 1:** Sociodemographic characteristics of the sample at T1 and T2 follow-up.

Characteristic		T1	T2
***N***		979	530
**Mothers’ age (years)**	*M (SD)*	31.97 (5.08)	32.54 (5.05)
	*Mdn*	32	33
	Range	18–48	18–48
**Target child’s age (years)**	*M (SD)*	2.43 (1.39)	2.52 (1.41)
	*Mdn*	3.0	3.0
	Range	0–5	0–5
**Target child’s sex**	Boys	49%	48%
	Girls	51%	52%
**Education**	Primary school	2%	2%
	High school	41%	34%
	Bachelor’s degree	18%	18%
	Master’s degree or above	39%	47%
**Living place**	Rural area	34%	34%
	City under 50,000 residents	17%	15%
	City between 50,000–100,000 residents	15%	14%
	City between 100,000–500,000	20%	22%
	City above 500,000 residents	15%	15%
**Employment**	Yes	71%	76%
	No	29%	24%
**Relationship status**	In relationship	93%	93%
	Single	7%	7%

At the 6-month follow-up (T2), all mothers participating at T1 were invited to participate again and completed a set of measures, including PRFQ. Of the baseline sample, 535 (55%) mothers responded. Nevertheless, individuals whose target child was over five years old at T2 were excluded (*n* = 5), and the final sample at T2 was *N* = 530. The frequency distribution of mothers based on children’s age was as follows: children below one year of age, 12%; 1-year-olds, 14%; 2-year-olds, 20%; 3-year-olds, 24%; 4-year-olds, 27%; 5-year-olds, 3% (see Table 1 for more sociodemographics). Post-hoc power analysis using a web-based sample size calculator for reliability [32, 33] revealed that our test–retest sample size was sufficient to detect a minimum acceptable ICC of .50, with an expected ICC of .70, α = .05, precision (± expected) = .05, two measurements (*k)* and expected drop-out rate of 10% (estimated minimum *n* = 446).

We applied purposive and volunteer sampling with the following inclusion criteria: (a) age above 18 years, (b) Polish nationality, and (c) being a biological mother of a child aged between 0 and 5 years. The exclusion criteria were as follows: (a) having a child with any known developmental delay or disability or significant sensory deficits such as blindness or hearing deficits (b) suicidal ideation or self-report of past or current psychiatric disorders (e.g., bipolar disorder, schizophrenia).

#### Procedure

Mothers were recruited online through the National Research Panel Ariadna, which is a Polish online survey panel accredited by the Interviewer Quality Control Programme (Polish: *Program Kontroli Jakości Pracy Ankieterów*; PKJPA) and conformed to the ICC/ESOMAR International Code for Market and Social Research standards. During registration in the Ariadna, the respondents accepted the panel’s terms and conditions and agreed to receive invitations to participate in the research. The Ariadna randomly recruited participants from among its 1.5 million registered users. Written informed consent was obtained in two steps. First, the panel obtained general online informed consent from participants, ensuring that they understood and accepted the panel’s terms and conditions (by checking the agreement field) and compliance with the RODO protocols. Next, we independently provided subjects with the information they needed to decide whether to volunteer for the present study. The information included: a statement that the project is research and participation is voluntary and free to terminate at any time; a summary of the research (including its purpose, duration, and list of procedures, potential discomforts, and expected benefits); a statement that results from this study may be published or presented at a research conference; and the contact to the principal investigator in case of any questions or comments. Written informed consent was obtained from the participants by clicking the checkbox in the statement: "I am at least 18 years of age, and I agree to participate in the research described above". Once a participant selected all the buttons, she was directed to the research survey questionnaire. At study entry, mothers were instructed to select one target child aged 0–5 years for whom to respond. They were then asked to complete all child-related items based on that child. The Ariadna panel accumulated the data and provided the data file for analysis, which was free of any identifiers (IP addresses or other electronic identifiers). The follow-up study (T2) followed the same format and procedures as those at T1.

As mentalizing (and thus PRF) is sensitive to stress and emotion arousal [13, 17], we intended to minimize the possible confounding effects of other measures on PRFQ scores. For instance, attachment measures might activate the participant’s attachment system, or reflection on parenting stress and psychopathology symptoms might increase anxiety. Therefore, we applied the measures in the following fixed order: sociodemographic factors, parental reflective functioning, attachment, childrearing stress and role restrictions, depressive symptoms, and BPD symptoms.

### Measures

The instruments we selected to measure the criterion validity of the PRFQ were also used in other similar studies on PRFQ (e.g., [1, 19, 29, 34, 35]). Using these measures allowed us to compare our results with those obtained in other studies that confirmed the validity of the PRFQ. The sociodemographic scale was included to explore the links between SES characteristics and PRFQ subscales, as these features may influence PRF [1]. The developers of each measure in this study have approved their use in this research.

#### Polish version of the Parental Reflective Functioning Questionnaire (PRFQ)

The PRFQ consists of 18 items divided into three subscales. First, the prementalizing (PM) subscale focuses on parents’ challenges in accurately perceiving their child’s mental states and their tendency to make inaccurate and hostile attributions toward their children (e.g., "My child sometimes gets sick to keep me from doing what I want to do"). High scores on this scale indicate serious distortions in PRF. Second, the certainty about mental states (CMS) subscale assesses the extent to which parents recognize the opacity of mental states and believe that they understand their children’s mind (e.g., "I always know what my child wants"). Scores on this scale may range from a tendency to be overly certain about the mental states of their child (hypermentalizing) to an almost complete lack of certainty about the child’s mental states (hypomentalising). The third subscale is interest in and curiosity about mental states (IC; e.g., "I am often curious to find out how my child feels"). Low scores potentially reflect an absence of interest in the child’s mental states, but very high scores potentially indicate intrusive hypermentalizing. Each subscale comprises six items which are scored on a 7-point Likert scale ranging from *strongly disagree* (1) to *strongly agree* (7). The original scale was proved to have good validity [22, 26] and internal consistency for all subscales, with Cronbach’s α ranging from .70 to .82 [1].

*Translation of the PRFQ*. As the first step in the Polish adaptation process, we obtained permission to translate the PRFQ from the main author (PL). We then translated the original English PRFQ using a standard back-translation procedure [36]. Initially, a professional team of four psychologists from the Center for Research on Personality Development at SWPS University (Poland) translated the PRFQ into Polish. In the translation, we regarded the original wording of the measure, but we also included its conceptual character. The researchers synthesized the versions produced by each psychologist into one version. Then, two linguistic professional independent translators performed the back-translation into English. These two back-translations were synthesized into one back-translated version of the PRFQ. Next, the expert group compared and interpreted the linguistic and cultural differences between the versions and assessed the questionnaire’s clarity, appropriateness, and equivalence. No significant discrepancies were found. Finally, we conducted an online pilot study among *N* = 309 mothers of children aged 0–5 years to assess the preliminary factor structure of the initial Polish version of the PRFQ (for details, see S1 Appendix in *Supporting Information*). Thereupon, we have made some further minor linguistic corrections in our translation (S1 Appendix). Data on the psychometric parameters of the PRFQ are provided in a subsequent section.

#### Experience in Close Relationship Scale-Revised and Shortened (ECR-RS)

We examined maternal attachment using the Experience in Close Relationship-Revised and Shortened Scale (ECR-RS) [37] in the Polish version by Lubiewska et al. [38]. The scale consists of 16 items assessing two dimensions underlying adult attachment: attachment anxiety, i.e., fear of rejection and abandonment (8 items, e.g., “I worry a lot about my relationships”) and attachment avoidance, i.e., discomfort with closeness and discomfort with depending on others (8 items; e.g., “I prefer not to be too close to romantic partners”). Participants rate the extent to which each item accurately describes them using a 7-point scale ranging from 1 (strongly disagree) to 7 (strongly agree). Two scores were computed by averaging items on each subscale after appropriately reverse scoring some items. The scale has established validity and good reliability (α = .89; ω = .77 for the anxiety dimension and α = .81; ω = .80 for the avoidance dimension) [38]. In this study, ω = .92, α = .92 for anxiety and ω = .90, α = .89 for avoidance.

#### Maternal childrearing stress and perceived role restrictions scales

We assessed maternal childrearing stress and perceived role restrictions using two brief scales created by Piotrowski [39] and based on the work of Ponnet et al. [40] and Van den Troost [41]. The Childrearing Stress Scale measures mothers’ perceptions of childrearing as burdensome and problematic and consists of 3 items (e.g., “Raising my child is much more difficult than I expected”). The Perceived Role Restrictions Scale assesses the degree to which a mother perceives parenthood as limiting and preventing her from living the lifestyle she would like to have. This scale includes four items (e.g., “Raising my child prevents me from doing what I like”). Each item was assessed on a five-point Likert scale, ranging from 1 (strongly disagree) to 5 (strongly agree). The two scores were obtained by averaging the item scores so that the higher scores reflect higher levels of childrearing stress and parental role restriction. In the original study, α = .81 for the childrearing stress dimension and α = .88 for role restriction, and CFA indicated its two-factor structure [16]. In the present study, ω = .85, α = .84 for the childrearing stress scale and ω = .91, α = .91 for role restrictions.

#### Epidemiological Studies Depression Scale-Revised (CESD-R)

Depressive symptoms were assessed using the Polish version of the Centre for Epidemiological Studies Depression Scale-Revised (CESD-R) [42, 43]. This self-report scale assesses the prevalence of affective symptoms, particularly depressive mood. The measure consists of 20 items rated on a scale from 0 (not at all or less than one day) to 4 (nearly every day for two weeks). In this study, the average of items indicated the level of depressive symptoms, with higher scores reflecting higher depressive symptoms. The scale has good validity and reliability (α = .95) [43]. In this study, we obtained the same result, ω = .95, α = .95.

#### Borderline personality disorder checklist (BPD checklist)

Maternal borderline symptoms were assessed using the BPD Checklist [44], a 47-item self-report questionnaire developed to assess the subjective burden caused by BPD symptoms during the last month. The items were based on the DSM-IV BPD criteria, the literature describing BPD manifestations, and clinical observations. Items are rated on a 5-point Likert scale, ranging from 1 (not at all) to 5 (extremely). The total score was computed by averaging the items on each subscale. The scale has good validity and excellent reliability (α = .0,97) [43]. In this study, we obtained the same values, ω = .97, α = .97.

#### Sociodemographic characteristics

We created a short sociodemographic questionnaire that included the mother’s age (years), child’s age (years), sex (boy; girl), maternal level of education, maternal employment status, family’s living area, and maternal partnership status. The questionnaire included a four-point list of alternatives to assess the level of education (i.e., primary school, high school, bachelor’s degree, master’s education or above), with a higher number indicating a higher education level. A two-point list of alternatives (i.e., yes; no) was used to assess maternal employment status and partnership status. The family’s living area was measured on a five-point scale, with a higher number indicating a locality with a higher number of inhabitants (i.e., rural area; city under 50,000 residents; city between 50,000–100,000 residents; city between 100,000–500,000; city with more than 500,000 residents).

### Ethical considerations

The American Psychological Association’s guidelines for good research practice and the Declaration of Helsinki were both followed throughout the study’s procedures. This study was approved by the Research Ethics Committee of the SWPS Faculty of Psychology and Law in Poznań (Date: July 24, 2021, reference no. 2021-97-12).

### Data analyses

We performed statistical analyses using the Jamovi 2.3 software [45]. We calculated the minimal sample size for CFA analysis using Kim’s [31] formula for noncentrality parameter calculation (*N*_min_ = 217 participants; for details see: *Sample* section). Before analysis, we examined data for outliers, i.e., responses outside possible ranges and absolute Z-score values greater than ±3 *SD* [46], missing data, and normality. We identified some outliers on the PM scale (*n* = 1) and the IC scale (*n* = 4). Furthermore, we identified some outliers among the criterion variables, i.e., borderline symptoms (*n* = 2), depressive symptoms (*n* = 1), and attachment avoidance (*n* = 1). After further inspection, we found that the outliers selected only the same extreme values on the given response scale, regardless of the item content. Therefore, we treated these outliers as potential sources of response-style-induced measurement errors and excluded them from further analyses. When we rerun the analyses with those outliers kept in the data, the results did not change. Because of the forced response, the database had no missing data. The final sample consisted of 979 participants.

For CFA purposes, we assessed the distribution normality for each PRFQ item by examining their skewness and kurtosis. The absolute value range of skewness was 0.17–1.28 (SE = 0.08), and the absolute value range of kurtosis was 0.13–3.44 (SE = 0.16), which allows the distributions to be considered close to normality [47]. We also assessed the multivariate normality of PRFQ items using Mardia’s multivariate normality test [48] through the SEMj module implemented in Jamovi 2.3 [45]. The analysis indicated that the data were not multivariate normal; Mardia’s kurtosis = 454.14, *p* < .001 and Mardia’s skewness = ;37.35, *p*< .001, *z* = 54.89, χ^2^(1140) = 6093.96, *p* < .001. Therefore, we performed CFA using maximum likelihood estimation with robust standard errors and a mean-and-variance adjusted (MLMV) as it yields the best combination of accurate standard errors and Type I errors for nonnormal data [35, 49].

We examined the hypothesized original factor structure as in Luyten et al. [1], followed by changes to the model based on model fit indices, nonsignificance of path coefficients, and substantive suggestions offered by modification indices. Standardized factor loadings of at least 0.40 were considered appropriate [50]. We evaluated model fit using the following indices [51–54]: model χ^2^, in which a nonsignificant value indicates an adequate fit (nevertheless, it should be noted that χ^2^ is almost always significant in large samples [55]); SRMR (standardized root mean square residual), in which a value lower than .08 indicates an adequate fit; RMSEA (the root mean square error of approximation), in which a value lower than .08 indicates an adequate fit and a value lower than .06 a good fit; CFI (comparative fit index) and NNFI (Bentler-Bonnet nonnormed fit index), for both of which a value higher than .90 indicates an adequate fit and a value higher than .95 a good fit.

Following the CFA, we assessed the internal consistencies of the Polish PRFQ subscales using Cronbach’s alpha (α) and McDonald’s omega (ω). Cronbach’s alpha and ω coefficients of .70 were considered to have at least good internal consistency [56, 57]. In addition, as this study was part of a broader longitudinal study (see *Funding* section), we were able to assess the six-month test–retest reliability in a subsample of mothers (*n* = 534; *M*_age_ = 32.54, *SD* = 5.05; range: 18–48 years old). For this purpose, we used test–retest data to calculate intraclass correlation coefficients (ICCs) and their 95% confidence intervals based on a single-measurement, absolute-agreement, two-way mixed-effects model. ICC values can indicate poor (< .50), moderate (≥ .50 to < .75), good (≥ .75 to < .90), and excellent (≥ .90) test–retest reliability [58].

Next, we calculated the descriptive statistics and conducted preliminary analyses. Formal normality tests, including the Shapiro–Wilk and Kolmogorov–Smirnov tests, may be unreliable for large samples and almost always yield significant deviations from normality at large sample sizes [59]. To check the univariate normality of particular variable scores, we applied the statistical method of skewness and kurtosis. We used the cut-off criteria suggested by George and Mallery [60] for skewness and kurtosis between -2 and +2 as reference values for determining nonsubstantial departure from normality. Therefore, the distributions of most of the continuous variables in our study are fairly symmetrical. The exception was the distribution of borderline symptoms with a kurtosis of 2.20, indicating a substantial violation of normality. In the next step, we assessed the links between Polish PFRQ subscales and sociodemographic continuous variables (i.e., child’s age and mother’s age) using Pearson’s *r* coefficient. We investigated the links between the PRFQ subscales and sociodemographic ordinal variables (i.e., mothers’ education and the size of the family’s living area) using Spearman’s *rho* coefficient. We assessed differences in PM, CMS, and IC levels regarding the child’s sex and maternal employment status using the Welsch’s t-test and Mann–Whitney’s U-test, respectively. We also compared the PRFQ scores within mothers who completed our 6-month test–retest follow-up with *t*-tests for dependent samples. We examined the linear links between PRFQ subscales and most criterion variables using Pearson’*s r* coefficient. The exception here was correlations with borderline symptoms. Because its distribution substantially deviated from normality, we used Spearman’s *rho*. To minimize the risk of a type I error when calculating numerous correlation coefficients [60], we applied the Bonferroni correction for multiple comparisons in a set of 30 correlation coefficients (significance set at *p* = .05/30). Therefore, results with values of *p* < .002 were considered statistically significant. In interpreting the correlation coefficients, we adopted the following criteria: coefficients ranging from 0 to 0.3 were considered to have a weak linear relationship, coefficients between 0.3 and 0.7 were considered to have a moderate relationship, and coefficients between 0.7 and 1.0 indicated a strong linear relationship [61].

Finally, we investigated the possibility of non-linear, U-shaped relationships between the CMS and IC subscales and each criterion variable. Because quadratic regression cannot be trusted to uncover a U-shaped or inverted U-shaped relationship because it elevates the risk of false-negative or false-positive findings [62, 63], we applied Simonsohn’s two-line test [78], which is a more robust alternative in testing U-shaped relationships. This test estimates an interrupted regression, i.e., a regression with two separate regression lines (slopes) for lower and higher values of a predictor hypothesized to have a u-shaped effect [62, 63]. In this test, the breakpoint is set using the "Robin Hood" algorithm, seeking to obtain higher power to detect a U-shape if it is present [62, 63]. If the resulting two slopes have opposite signs and are each statistically significant, the test rejects the null hypothesis that there is no U-shaped (nor inverted U-shaped) effect. These analyses were conducted using Simonsohn’s application for running the two-line test, available at http://webstimate.org/twolines [62]. Complete data for these analyses were available for the entire sample (*N* = 979).

## Results

### Demographics

Some significant differences were found between mothers who participated in follow-up and those who did not. Mothers participating in follow-up were slightly older (*Mdn* = 33.00) than those who did not (*Mdn* = 31.00), *U* = 100258.00, *p* < .001, *r*_*pb*_ = .14. Moreover, their target children were slightly older (*Mdn* = 3.00) than those of mothers who did not participate at T2 (*Mdn* = 2.00), *U* = 1007295.00, *p* = .03, *r*_*pb*_ = .08. Levels of education of mothers at T2 were higher (*Mdn* = 3.00) than those who participated only at T1 (*Mdn* = 2.00), *U* = 94022.00, *p* < .001, *r*_*pb*_ = .19. However, all these effects were small. Finally, more mothers were employed among participants in the follow-up study (*n* = 400) than among those who participated only at T1 (*n* = 290), χ2(1) = 10.71, *p* = .001. No other differences in terms of demographic features were found.

### Confirmatory factor analysis

**Fig 1:**
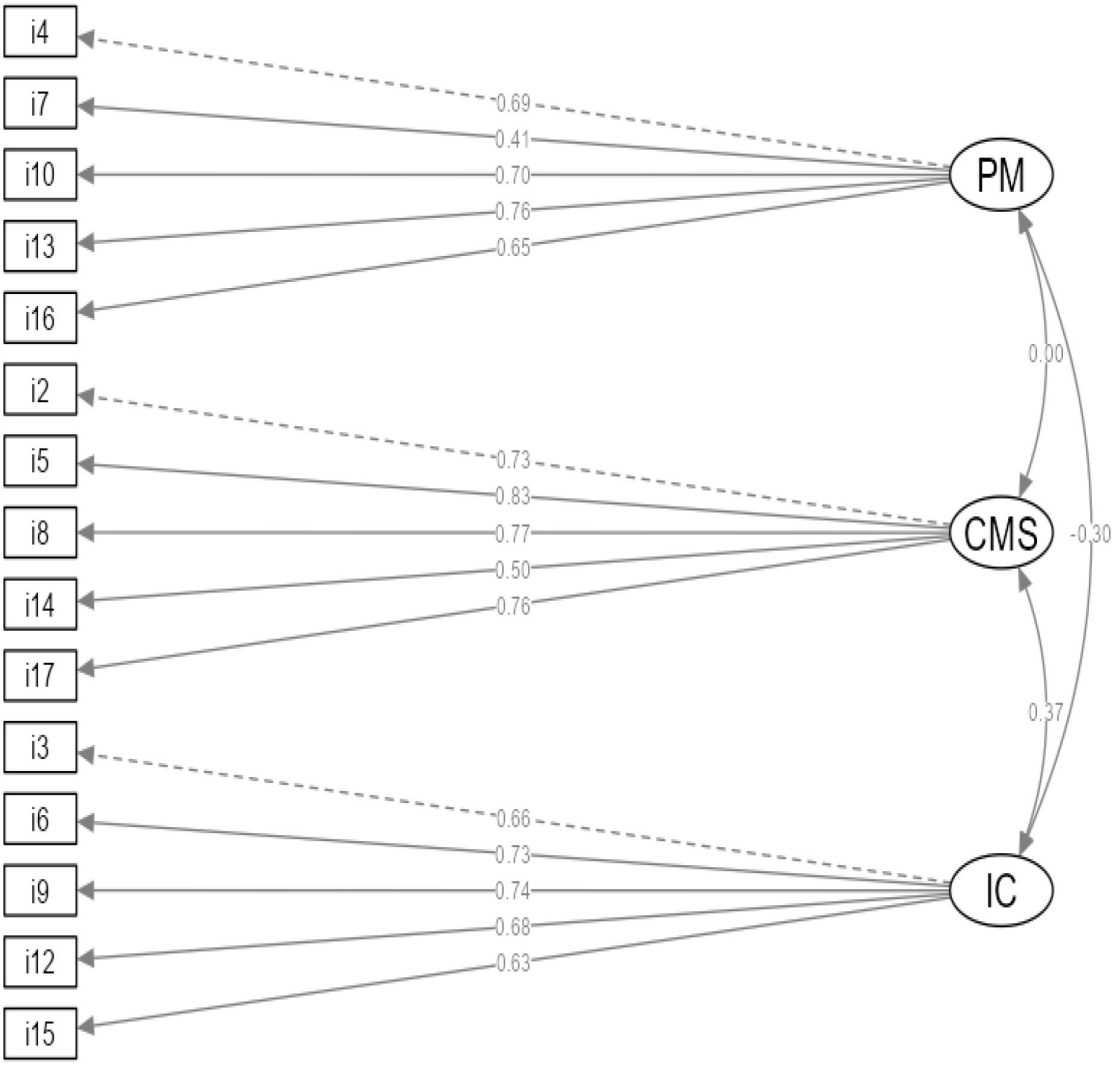
Three-dimensional factor structure of the PRFQ in mothers. *Note*. The final model without items 1, 11, and 18. Ellipses indicate factors, and rectangles indicate items. *St*andardized loadings are indicated by the numbers between the single-arrow lines. Relationships between the factors are implied by the number on the right between the bidirectional arrows. Residuals and correlations between residuals are omitted for clarity of presentation. All standardized regression weights of factor loadings were significant (*p* < .001).

Factor structure analysis of the Polish translation of the PRFQ resulted in the examination of five different CFA models (Table 2). The initial Model 1 testing of the original three-factor structure of the PRFQ (PM, CMS, and IC) [1] resulted in a poor model fit to the data, as shown in Table 2. The results indicated that item 11 had the weakest standardized factor loading (β = .22, *p* < .001) to the PRFQ measure. Therefore, we conducted a second CFA to verify whether or not the model fit could be improved by removing item 11. The results in Model 2 revealed improvements in the model fit statistics reported in Table 2. However, this model revealed a relatively low standardized factor loading for item 1 (β = 0.35, *p* < .001). Hence, we removed item 1 in Model 3 to further enhance the model fit. After testing Model 3, the results identified a more respectable model fit (Table 2); however, modification indices suggested that item 18 cross-loaded significantly onto the IC and PM factors. Thus, we have removed item 18 from Model 4. The results revealed further model-fit improvement (Table 2). In this model, modification indices suggested a better model fit by adding a covariance between the errors for items 6 and 9 and 14 and 17. Because these pairs of items belong to the same subscales, their measurement errors were allowed to correlate [52]. Adding these correlated error terms further improved the model fit, as shown in Table 2 (Model 5). Finally, with these modifications, a three-factor Polish version of the PRFQ that does not include items 1, 11, and 18 was identified as a good fit to the data (Fig 1). The Polish PRFQ consisted of 5 items per scale (see S2 Appendix), and, as shown in Table 3, all items had standardized factor loadings higher than .40 in the final model.

**Table 2:** Fit statistics for confirmatory factor analysis models (N = 979).

Model	χ^2^ (*df*)	SRMR	RMSEA [95% CI]	CFI	NNFI
**Model 1** ^a^	810.79 (132)	.10	.07 [.07-.08]	.71	.66
**Model 2** ^b^	589.97 (116)	.08	.06 [.06-.07]	.79	.75
**Model 3** ^c^	534.61 (101)	.08	.07 [.06-.07]	.80	.76
**Model 4** ^d^	321.51 (87)	.05	.05 [.05-.06]	.89	.87
**Model 5** ^e^	268.20 (85)	.05	.05 [.04-.05]	.91	.89

*Note*. 95% CI—95% confidence interval. SMRM—standardized root mean square residual; RMSEA—root mean square error of approximation; CFI—comparative fit index; NNFI–Bentler-Bonnet nonnormed fit index.

^a^ Initial model contains all 18 items.

^b^ Model 2 has item 11 removed.

^c^ Model 3 has items 1 and 11 removed.

^d^ Model 4 has items 1, 11, and 18 removed.

^e^ Model 5 adds covariance between the errors of items 6 and 9 and those of 14 and 17.

**Table 3:** Standardized factor loadings of the final Polish PRFQ and the reliability values (N = 979).

Factors	Item	Standardized factor loadings	Cronbach’s alpha (α)	Omega (ω)
**Prementalizing modes (PM)**	Item 4: *My child cries around strangers to embarrass me*	.68	.76	.76
	Item 7: *I find it hard to actively participate in make-believe play with my child*	.41		
	Item 10: *My child sometimes gets sick to keep me from doing what I want to do*	.70		
	Item 13: *When my child is fussy he or she does that just to annoy me*	.76		
	Item 16: *Often*, *my child’s behavior is too confusing to bother figuring out*	.64		
**Certainty about mental states (CMS)**	Item 2: *I always know what my child wants*	.74	.84	.82
	Item 5: *I can completely read my child’s mind*	.84		
	Item 8: *I can always predict what my child will do*	.77		
	Item 14: *I always know why I do what I do to my child*	.47		
	Item 17: *I always know why my child acts the way he or she does*	.74		
**Interest and curiosity in mental states (IC)**	Item 3: *I like to think about the reasons behind the way my child behaves and feels*	.67	.82	.79
	Item 6: *I wonder a lot about what my child is thinking and feeling*	.66		
	Item 9: *I am often curious to find out how my child feels*	.68		
	Item 12: *I try to see situations through the eyes of my child*	.71		
	Item 15: *I try to understand the reasons why my child misbehaves*	.65		

*Note*. Items removed: Item 1 *The only time I’m certain my child loves me is when he or she is smiling at me;* Item 11 *I can sometimes misunderstand the reactions of my child;* Item 18 *I believe there is no point in trying to guess what my child feels*.

Next, we calculated the subscales based on the final model. There was a weak negative correlation between PM and IC, *r*(977) = .23; *p* < .001, and a moderate positive correlation between IC and CMS, *r*(977) = .35, *p* < .001. On the other hand, PM and CMS were not correlated (*r*(977) = .04, *p* = .21), suggesting that the questionnaire measures three relatively independent characteristics of parental reflective functioning (Fig 1).

#### Internal consistency and test–retest reliability

As shown in Table 3, the PRFQ subscales showed good internal consistency, with both Cronbach’s α and McDonald’s ω values above 0.75 for the three subscales. The 6-month interval test–retest reliability (*n* = 534) of PM, CMS, and IC was moderate, with ICCs of .57 [95% CI = .51–.63], .68 [95% CI = .63–.72] and .60 [95% CI = .52–.67] respectively.

### Descriptive statistics and preliminary analyses

Descriptive statistics for the continuous variables at baseline (T1) are presented in the *Supporting Information* (S1 Table). As a part of the preliminary analyses, we investigated the relationship between the PRFQ subscales and continuous sociodemographic characteristics (Table 4). All correlations between PRFQ subscales and sociodemographic variables reflected small effect sizes, and several were no longer significant after correction for multiple comparisons. PM was slightly positively related to the child’s age. Furthermore, CMS was positively related to both the child’s and the mother’s age and negatively related to the mother’s level of education.

**Table 4:** Correlations among PRFQ dimensions and demographic characteristics (N = 979).

	Child age^a^	Mother age^a^	Size of the living area^b^	Level of maternal education^b^
**PM**	.11***	- .02	-.09	-.04
**CMS**	.16***	.12***	-.04	-.12***
**IC**	.08	-.01	.04	-.07

*Note*. PM—prementalizing modes; CMS—certainty about mental states; IC—interest and curiosity in mental states.

*** p < .002 (Bonferroni correction applied).

^a^ Correlations between this variable and the PRFQ scales were determined using Pearson’s *r*.

^b^ Correlations between this variable and the PRFQ scales were determined using Spearman’s *rho*.

There were no significant differences in the PRFQ dimensions regarding the child’s sex and maternal employment status (for details, see S2 and S3 Tables). As in our sample of mothers, the vast majority (93%; *n* = 908) were in a relationship; we did not assess differences in the three PRFQ scales regarding maternal partnership status. We also compared the PRFQ scores among mothers who completed the 6-month test-retest follow-up (*n* = 534). Descriptive statistics for the continuous variables at T2 are presented in the Supporting Information (S4 Table). The *t*-test for dependent samples revealed that PM scores were slightly higher in T2 than in T1, *t*(529) = -4.61, *p* < .001, Cohen’s *d* = -0.20. A similar increase was observed for IC scores, *t*(529) = -7.18, *p* < .001, Cohen’s *d* = -0.31. On the other hand, CMS scores did not differ significantly in the 6-month time interval, *t*(529) = 1.01, *p* < .31, Cohen’s *d* = -0.04.

### Criterion validity of the PRFQ

Table 5 presents the results of the linear correlational analysis between the PRFQ-PL-15 dimensions and the criterion variables. As expected, PM had a positive relationship with attachment dimensions, severity of psychopathology (severity depression and BPD features), childrearing stress, and maternal role restrictions. These correlations reflected small to medium effect sizes. Similarly, CMS was negatively associated with all the criterion variables, and all correlations were of small effect sizes. Finally, IC was negatively correlated with attachment avoidance and perceived role restrictions. However, we did not find any significant relationships between IC and the remaining criterion variables.

**Table 5:** Zero-order correlations between PRFQ subscales and criterion variables (N = 979).

	PM	CMS	IC
**Attachment anxiety** ^a^	.35***	-.12***	-.06
**Attachment avoidance** ^a^	.29***	-.19***	-.21***
**Childrearing stress** ^a^	.22***	-.29***	.01
**Role restrictions** ^a^	.35***	-.29***	-.12***
**Borderline symptoms** ^b^	.39***	-.18***	-.05
**Depressive symptoms**	.30***	-.14***	.04

*Note*. PM—prementalizing modes; CMS—certainty about mental states; IC—interest and curiosity in mental states.

*** p < .002 (Bonferroni correction applied).

^a^ Correlations between this variable and the PRFQ scales were determined using Pearson’s *r*.

^b^ Correlations between this variable and the PRFQ scales were determined using Spearman’s *rho*.

There was no evidence for nonlinear relationships between the PRFQ subscales and criterion variables (S5 Table in *Supporting materials*).

## Discussion

This study aimed to evaluate the psychometric parameters of the Polish version of the Parental Reflective Functioning Questionnaire (PRFQ) [1] and examine whether the scale validly measures this aspect of parental mentalization among Polish mothers of infants and young children. Our results confirm that with minor modifications to the original version of the PRFQ, the Polish adaptation can be considered a valid and reliable parental mentalization scale.

### Factor structure

CFA confirmed the three-factor structure of the original PRFQ. However, item 1 (“*The only time I’m certain my child loves me is when he or she is smiling at me*”; PM subscale), item 11 (“*I can sometimes misunderstand the reactions of my child*”; reverse coded, CMS subscale), and item 18 (“*I believe there is no point in trying to guess what my child feels*”; reverse coded, IC subscale) showed low and nonsignificant loadings. The results regarding the low loadings of items 11 and 18 are consistent with previous research on Canadian [19], Hungarian [22], Chinese [23], Italian [29], and American [30] adaptations of PRFQ. One reason for these findings might be that those items are reverse-worded; thus, their meanings may be more easily misunderstood, and therefore, they may not measure the same underlying general trait [19, 23, 64]. Moreover, the original wording of item 11 requires a mother to reflect on her certainty about her internal mental states (as opposed to her certainty about her child’s mental states), and the answers may depend on the mother’s reflective and metacognitive capacities. A similar remark was made by Edler et al. [30]. The original wording of item 18 is opposed to the remaining items on the IC scale, as they show cognitive curiosity and attentive sensitivity to the *child’s* needs. Moreover, it emphasizes whether a mother thinks it is valuable to guess what the child is feeling rather than directly indicating whether she is interested in her child’s feelings. As Edler et al. [30] note, mothers who question their parenting abilities may answer these items differently than those who directly refer to their children’s mental states. Nevertheless, modification of the wording of this item did not improve its factor parameters.

The three factors of the PRFQ were, as in other studies, only weakly related, which is in line with the notion that the PRFQ is a multidimensional construct with relatively distinct aspects [1, 3]. On the other hand, the observed pattern of links between the PRFQ subscales might also have resulted from the nature of our nonclinical sample. In our study, the mean maternal PM value was relatively low (*M* = 2.22, *SD* = 1.05, *Q3* = 2.80), indicating that our sample was quite homogenous regarding PM and had relatively low difficulties in accurately perceiving their child’s mental states. This low variability in PM scores may be the cause of the independence between PM and more heterogeneous CMS scores.

### Reliability

The Cronbach’s alpha and McDonald’s omega coefficients of the PRFQ factors ranged from .76 to .87, indicating good reliability for each of the three subscales of the PRFQ. CMS and IC are consistent with other studies [20, 21, 23, 24, 29]. On the other hand, in some of them, Cronbach’s alphas for PM are lower than those for the remaining subscales [20, 21, 23, 24, 29]. In the Polish PRFQ, the PM subscale had a relatively higher Cronbach’s alpha than some other PRFQ adaptations. As mentioned in the previous section, mothers with very high scores on PM were underrepresented. With no severe mentalizing difficulties, participants probably responded more consistently to the items on the PM scale, which operationalized a nuanced construct (for details, see [13]). As a parent with impairments in RF can display one aspect of PM but not another, their answers on the PM scale can be less correlated. This interpretation is consistent with other studies that revealed relatively lower PM scale reliability in clinical samples (e.g., [34]) compared with some normative samples (e.g., [1, 24]). Furthermore, certain items may resonate differently or be interpreted in distinct ways by diverse populations and participants from different cultures. Moreover, some presentation-bias might be involved in some samples and countries. However, these issues require further research.

The 6-month test–retest ICCs of the PRFQ subscales (*n* = 534) were all moderate, consistent with the notion that PRF has both trait and state features. Thus, several factors, such as parental stress and child features, influence PRF levels [65]. This means that it can fluctuate over time, depending on the level of emotional arousal, information processing mechanisms, change in a person’s self-evaluation, the stage of the child’s development, etc. This means that it can fluctuate over time, depending on the level of emotional arousal [13], information processing mechanisms, change in a person’s self-evaluation [66], and in response to child features and developmental demands more generally [1, 13, 29]. Longitudinal studies with different time intervals are required to further investigate this issue.

#### PRFQ and sociodemographic characteristics

There were no significant associations between the PRFQ dimensions and the child’s sex and maternal employment status. On the other hand, the PM subscale was positively correlated with the child’s age. We also found that CMS was positively related to the child’s and mother’s ages and negatively related to the mother’s level of education. However, regarding the small magnitudes of all these links, they were significant because of our large sample size. Therefore, we conclude that the PRFQ is relatively independent of sociodemographic features. This agrees with the results from most other countries (e.g., [1, 28, 29, 67, 68]), and it suggests that PRFQ can be used in socially diverse samples of mothers of young and preschool children.

### Criterion-related validity

Our findings revealed weak and negative linear links between CMS and both attachment anxiety and attachment avoidance. IC was also weakly and negatively correlated with attachment avoidance. On the other hand, the IC subscale was independent of attachment anxiety. All these links were of small magnitude, aligning with the concept of *loose coupling*, which claims that indices of more adaptive PRF (in the present study–IC especially) seem relatively unrelated to attachment [1, 69]. In contrast, PM was moderately positively linked to attachment anxiety and weakly positively relatesd to attachment avoidance. This agrees with the findings that insecure attachment tends to be related to more severe impairments in PRF, as expressed by higher PM levels [1, 69].

PM was linearly weakly and positively related to childrearing stress and moderately positively related to maternal perceptions of role restrictions. These findings agree with studies revealing that mothers with limited mentalizing abilities may be more susceptible to stressors as they fail to effectively cope with the demands of parenting [1, 19, 21, 23, 68]. Everyday challenges make them feel more overwhelming, which contributes to increased stress and limited ability to reconcile everyday duties and different life roles. Weak and negative links between CMS and childrearing stress and perceived role restrictions correspond to other studies suggesting that mothers with high CMS tend to believe that they can always identify and understand their child’s mental states properly [21, 23]. Therefore, they are likely to be confident in their parenting abilities, regardless of the accuracy of their representations, and they might experience less parenting stress and role restriction than mothers with relatively low CMS. IC was linearly weakly and negatively linked to perceived role restrictions, suggesting that higher IC might lead to more satisfaction from parenting, as indicated by the lower perceived constraints caused by having a child.

Analyses revealed that PM was moderately and positively linked to depressive and BPD symptoms. Moreover, depressive and BPD symptoms were negatively and weakly related to CMS. In contrast, IC was independent of psychopathological symptoms. Our findings are consistent with those of some other studies [21, 34, 70], suggesting that maternal depressive symptoms do not necessarily impact her curiosity in and active willingness to understand the child’s mental states. Our observations are also in line with studies indicating that depression can lead to cognitive distortions and a preoccupation with one’s emotional distress, making it difficult to attune to and understand a child’s emotional needs [24, 34, 35, 71, 72]. However, it should be noted that these links are likely bidirectional. It was also found that mothers who can effectively understand and respond to their children’s emotions may experience greater satisfaction from their parenting role, potentially reducing the risk of developing depressive symptoms [34]. The effect sizes of our findings are also congruent with those of the aforementioned studies..

The pattern of correlations between PRF and the symptoms of BPD is in line with the findings of Marcoux et al. [73] that mothers with and without BPD are equally likely to be interested and curious about mental states of their children. However, mothers with BPD appear to be more likely to misread their infants’ mental states. Hestbaek et al. [74] observed that parents with personality disorders reported higher PM and lower CMS levels than healthy controls.

In conclusion, the significant correlations between the three factors of the PRFQ and maternal attachment, parenting stress, maternal perceptions of role restrictions, depression, and borderline symptoms described above supported the empirical validity of the PRFQ-PL.

### Implications and limitations

This study has several limitations that should be noted. First, our sample included only mothers; future studies should include fathers. Including them would extend the generalization of the results to fathers and allow the analysis of sex invariance of the PRFQ. Moreover, it should be emphasized that most mothers in our sample had children aged 0–4, and mothers of 5-year-olds were underrepresented. Thus, further studies, ensuring equal frequencies of mothers regarding their children’s age, are needed to check the equivalence of PRFQ in samples of mothers of children aged 5 years. Until then, the results of the validation of PRFQ should be interpreted with caution in the case of mothers of five-year old children.

Regarding the fact that PRF fosters numerous children’s developmental outcomes, future studies on the PRFQ should include not only mothers’ data but also some child variables such as, e.g., attachment security [75], the capacity of affect regulation and psychosocial functioning [11], or children’s mentalizing capacity [9]. This would allow researchers to explore the validity of the PRFQ in more detail.

Another limitation of this study is that all measures used were based on self-report questionnaires. Thus, the findings of this study may partially reflect shared method variance. Using observational methods, such as attachment or mentalization narratives, would reveal a more complex picture of PRFQ validity in future studies. Moreover, in Poland, there is no alternative measure of PRF that could be used in an online study. Thus, we could not check the convergent validity of the PRFQ with other measures of PRF. Therefore, future offline studies should include traditional assessment of PRF typically involving interviews or narratives such as the Parental Development Interview (PDI), the Working Model of Child Interview (WMCI), and the Adult Attachment Interview (AAI) [12, 18]. However, measures of *general* reflective functioning might also be included.

Regarding the generalizability of our findings, the participating mothers were relatively homogeneous in terms of age and educational level; only 2% of the sample completed a lower than a high school education, and 34% of our sample lived in rural areas. In other words, the mothers were primarily highly educated, working, and living in urban areas. Thus, the results may not be generalizable to the general population. In addition, our study included a community sample of mothers; thus, the results are not generalizable to clinical or high-risk samples. Findings from these samples may be helpful for researchers and practitioners in evaluating parental reflective functioning processes, e.g., regarding the effectiveness of some clinical interventions. Further studies on the validity of PFRQ-15-PL with more disadvantaged samples are required.

Finally, participants in this study were recruited from the online survey panel; thus, the research is limited to internet users. Indeed, online recruitments are fraught with self-selection bias, e.g., participants tend to be more interested in the research problem and, thus, more motivated to participate [76]. Web panels may also attract low-income respondents because participation is rewarded with gifts [77]. Regarding this issue, future research could replicate these findings in a random sample of Polish mothers recruited through other means than online research.

### Conclusion

This study serves as a preliminary validation of the Polish version of the PRFQ. A three-factor structure with 15 items for the Polish version of the questionnaire (PRFQ) was supported, which had good internal consistency, moderate test–retest reliability, and was significantly correlated in theoretically predicted ways with maternal attachment, childrearing stress, perceived role restrictions, and depressive and borderline symptoms. These findings suggest that PRFQ can serve as a valid and reliable measure in Polish mothers of children aged 0–5 in nonclinical settings.

## Supporting information

S1 Appendix detailed information about the pilot study and further improvements in Polish translation of the PRFQ. (DOCX)

S2 Appendix The 15-item Polish version of the PRFQ (PRFQ). (DOCX)

S1 Table Descriptive statistics for the PRFQ subscales and the other study criterion variables (*N* = 979). Note. PM—prementalizing modes; CMS—certainty about mental states; IC—interest in and curiosity about mental states.
(DOCX)

S2 Table Welsch’s t-tests assessing maternal PRFQ subscales levels regarding child’s sex (*N* = 979). Note. PM—prementalizing modes; CMS—certainty about mental states; IC—interest in and curiosity about mental states.
(DOCX)

S3 Table Mann-Whitney U-tests assessing maternal PRFQ subscales levels regarding maternal employment status (N = 979). Note. PM—prementalizing modes; CMS—certainty about mental states; IC—interest in and curiosity about mental states.
(DOCX)

S4 Table Descriptive statistics for PRFQ scores after 6-month follow-up (*n* = 534). Note. PM—prementalizing modes; CMS—certainty about mental states; IC—interest in and curiosity about mental states.
(DOCX)

S5 Table Simonsohn’s two-lines test assessing U-shaped relationships between the criterion variables and maternal interest and curiosity in mental states and certainty about mental states (N = 979). Note. CMS—certainty about mental states; IC—interest in and curiosity about mental states. X—predictor; Y—dependent variable. Slope 1—regression line for lower values of a predictor; Slope 2 –regression line for higher values of a predictor. Breaking point—the value of X, which is the breaking point for the interrupted regression resulting in the two slope.
(DOCX)
