# Supplementary material for: Psychometric evaluation of the Parental Reflective Functioning Questionnaire in Polish mothers
Source: PLoS One. 2024 Apr 17;19(4):e0299427. doi: 10.1371/journal.pone.0299427 (PMC11023587; doi:10.1371/journal.pone.0299427)
Supplement: S1 Appendix — (DOCX) [file pone.0299427.s001.docx]

After the back-translation procedure (see *Measures* section in the paper), the next step was to assess the preliminary factor structure of the initial Polish version of the PRFQ. Therefore, we conducted an online pilot study on the sample of 309 mothers (age range: 18-50; *M* = 34.07, *SD* = 7.42) of children aged 0-5 years (*M* = 2.5, *Mdn* = 3.0, *SD* = 1.80), 50% of whom were boys. We conducted CFA with MLMV estimation with the same assumptions as CFA in our main study ( for details, see the Data Analyses section). CFA revealed poor model fit (χ^2^(132) = 329.26, p < .001, SRMR = .12, RMSEA = .07 (CI [.06, .08]); CFI = .67, NNFI = .62. Furthermore, there were relatively low factor loadings for item 1 (β = .32, p < .001), item 7 (β = .42, p < .001), and item 11 (β = .12, n.s.). Moreover, modification indices suggested that item 18 (CMS scale) was cross-loaded on the PM scale at. 84, and item 11 (also CMS scale) was cross-loaded on the IC scale at .58. Thus, we checked those items and adjusted them to make them easier for the participants to read and understand.

The original phrasing of item 1 (“The only time I’m certain my child loves me is when he or she is smiling at me”) was modified as follows: “Only when my child smiles at me can I be sure that she loves me.” As reading item 7 (“I find it hard to actively participate in make-believe play with my child”), some mothers might not know what “make-believe play” is; we added an explanation within the item as follows: “I find it difficult to actively participate in pretend play (i.e. in which someone pretends to be something) with my child.”

Regarding item 11 (“I can sometimes misunderstand the reactions of my child”; reverse-coded), all the other items in the CMS scale indicate the mother’s high confidence (“always”) in understanding her child’s mental states. Meanwhile, item 11 is the only one that indicates some doubt (“I can sometimes […]”), indicating relatively moderate than low (after reverse-coding) CMS levels. It seems that mothers who mentalize well understand that sometimes they misunderstand their children, but this can entail interest and curiosity even more. Finally, the English “I can sometimes” in Polish might have somewhat different syntax and thus semantic connotations than English. Thus, it might be misunderstood by Polish mothers. The issues mentioned above might be the cause of why item 11 was more strongly associated with IC than CMS. Therefore, we decided to reformulate item 11 to eliminate its content uncertainty. Hence, the new wording of the item was as follows: “I can never understand why my child behaves in a certain way”, which, after reverse-coding, would potentially clearly express the mother’s certainty about her child’s mental states.

Finally, item 18 (“I believe there is no point in trying to guess what my child feels”; reverse coded; originally in IC scale, cross-loading PM scale) has negative connotations due to the wordings "there is no point" and “trying to guess.” Moreover, it should be noted that the other items in the IC scale depict what the mother does, as opposed to item 11, which describes what a mother thinks about interest and attentive sensitivity to the child’s needs. Such formulation forces the mother to reflect on her opinion, which necessitates the involvement of metacognitive processes. Considering this, we decided to make item 11 more congruent with the rest of the IC scale by reflecting the mother’s behavior rather than her opinion. Hence, we modified item 18: "I don’t try to determine how my child feels in different situations." The latter is the most significant change we made to the original English version of PRFQ.

We have introduced all the above-mentioned changes to facilitate the reception and comprehensiveness of the questionnaire. To check the comprehensibility and feasibility of this new version of the Polish PRFQ translation, we consulted eight mothers (*M*_age_ = 30.12 years, *SD* = 3.04; age range: 26 – 35) of children aged 0-5, recruited from local nursery and kindergarten. These mothers did not raise any objection to the questionnaire and confirmed the clarity of the items. Therefore, we made no other changes after the pilot study and reached a consensus to finalize the revised manuscript. In the validation study, we used this version of the Polish translation of the PRFQ.
