# Supplementary material for: Psychometric evaluation of the Parental Reflective Functioning Questionnaire in Polish mothers
Source: PLoS One. 2024 Apr 17;19(4):e0299427. doi: 10.1371/journal.pone.0299427 (PMC11023587; doi:10.1371/journal.pone.0299427)
Supplement: S2 Appendix — (DOCX) [file pone.0299427.s002.docx]

**Factor 1: Prementalizing modes (PM)**

item 4: Moje dziecko płacze przy obcych ludziach, żeby mnie zawstydzić (My child cries around strangers to embarrass me)

item 7: Jest mi trudno aktywnie uczestniczyć w zabawie „na niby” (tj. w której ktoś coś udaje) z moim dzieckiem (I find it hard to actively participate in make-believe play with my child)

item 10: Moje dziecko czasem choruje po to, żeby powstrzymać mnie od robienia tego, co chcę (My child sometimes gets sick to keep me from doing what I want to do)

item 13: Kiedy moje dziecko marudzi, to robi to po prostu po to, żeby mnie zirytować (When my child is fussy he or she does that just to annoy me)

item 16: Często zachowanie mojego dziecka jest zbyt zagmatwane, aby zawracać sobie głowę jego rozgryzaniem (Often, my child’s behaviour is too confusing to bother figuring out)

**Factor 2: Certainty about Mental States (CMS)**

item 2: Zawsze wiem, czego chce moje dziecko (I always know what my child wants)

item 5: Zawsze dokładnie wiem, co myśli moje dziecko (I can completely read my child’s mind)

item 8: Zawsze potrafię przewidzieć, co zrobi moje dziecko (I can always predict what my child will do)

item 14: Zawsze wiem dlaczego tak, a nie inaczej zachowuję się wobec mojego dziecka (I always know why I do what I do to my child)

item 17: Zawsze wiem, dlaczego moje dziecko zachowuje się tak, jak się zachowuje (I always know why my child acts the way he or she does)

**Factor 3: Interest and Curiosity in Mental States (IC)**

item 3: Lubię zastanawiać się nad przyczynami stojącymi za tym, jak zachowuje się i co czuje moje dziecko (I like to think about the reasons behind the way my child behaves and feels)

item 6: Dużo zastanawiam się nad tym, co myśli i czuje moje dziecko (I wonder a lot about what my child is thinking and feeling)

item 9: Często ciekawi mnie by dowiedzieć się, co czuje moje dziecko (I am often curious to find out how my child feels)

item 12: Próbuję zobaczyć różne sytuacje oczami mojego dziecka (I try to see situations through the eyes of my child)

item 15: Staram się zrozumieć powody, dla których moje dziecko źle się zachowuje.

(I try to understand the reasons why my child misbehaves)

**Removed items:**

item 1 (PM): Tylko wtedy gdy moje dziecko uśmiecha się do mnie, mam pewność, że mnie kocha (The only time I’m certain my child loves me is when he or she is smiling at me)

item 11 (CMS): Nigdy nie potrafię zrozumieć, dlaczego moje dziecko zachowuje się w określony sposób (I can sometimes misunderstand the reactions of my child) [Notice that in the original PRFQ, item 11 is reverse-coded, but this is not the case in the Polish version of the measure (see manuscript for details)].

item 18 (IC): Nie próbuję dowiadywać się co czuje moje dziecko w różnych sytuacjach (I believe there is no point in trying to guess what my child feels) [reverse coded].
