# Supplementary material for: Psychometric evaluation of the Parental Reflective Functioning Questionnaire in Polish mothers
Source: PLoS One. 2024 Apr 17;19(4):e0299427. doi: 10.1371/journal.pone.0299427 (PMC11023587; doi:10.1371/journal.pone.0299427)
Supplement: S1 Table — Note. PM—prementalizing modes; CMS—certainty about mental states; IC—interest in and curiosity about mental states. (DOCX) [file pone.0299427.s003.docx]

| **Variable** | ***M*** | ***SE*** | ***Mdn*** | ***SD*** | **Range** | **Minimum** | **Maximum** | **Skewness** | ***SE*** | **Kurtosis** | ***SE*** |
| --- | --- | --- | --- | --- | --- | --- | --- | --- | --- | --- | --- |
| **PM** | 2.22 | 0.03 | 2.00 | 1.05 | 5.60 | 1.00 | 6.60 | 0.94 | 0.08 | 0.54 | 0.16 |
| **CMS** | 4.22 | 0.04 | 4.20 | 1.23 | 6.00 | 1.00 | 7.00 | -0.20 | 0.08 | -0.45 | 0.16 |
| **IC** | 5.33 | 0.03 | 5.40 | 1.01 | 5.60 | 1.40 | 7.00 | -0.47 | 0.08 | 0.07 | 0.16 |
| **Attachment anxiety** | 3.42 | 0.05 | 3.38 | 1.56 | 6.00 | 1.00 | 7.00 | 0.14 | 0.08 | -1.02 | 0.16 |
| **Attachment avoidance** | 2.49 | 0.04 | 2.38 | 1.11 | 5.63 | 1.00 | 6.63 | 0.72 | 0.08 | 0.21 | 0.16 |
| **Childrearing stress** | 3.17 | 0.03 | 3.33 | 1.05 | 4.00 | 1.00 | 5.00 | -0.26 | 0.08 | -0.70 | 0.16 |
| **Role restrictions** | 2.59 | 0.03 | 2.50 | 1.07 | 4.00 | 1.00 | 5.00 | 0.25 | 0.08 | -0.75 | 0.16 |
| **Borderline symptoms** | 1.67 | 0.02 | 1.49 | 0.58 | 3.79 | 1.00 | 4.79 | 1.41 | 0.08 | 2.20 | 0.16 |
| **Depressive symptoms** | 0.88 | 0.03 | 0.65 | 0.78 | 4.00 | 0.00 | 4.00 | 1.14 | 0.08 | 0.82 | 0.16 |
| **Mother’s age** | 31.97 | 0.16 | 32.00 | 5.08 | 30.00 | 18.00 | 48.00 | 0.05 | 0.08 | -0.16 | 0.16 |
