# Supplementary material for: Psychometric evaluation of the Parental Reflective Functioning Questionnaire in Polish mothers
Source: PLoS One. 2024 Apr 17;19(4):e0299427. doi: 10.1371/journal.pone.0299427 (PMC11023587; doi:10.1371/journal.pone.0299427)
Supplement: S2 Table — Note. PM—prementalizing modes; CMS—certainty about mental states; IC—interest in and curiosity about mental states. (DOCX) [file pone.0299427.s004.docx]

|  |  |  |  | **Boys  (*n* = 480)** | **Girls (*n* = 499)** |  |
| --- | --- | --- | --- | --- | --- | --- |
|  | ***t*** | ***df*** | ***p*** | ***M (SD)*** | ***M (SD)*** | **Cohen’s *d*** |
| **PM** | 0.09 | 973.26 | .93 | 2.21 (1.06) | 2.22 (1.04) | 0.01 |
| **CM** | 0.96 | 964.68 | .34 | 4.19 (1.27) | 4.26 (1.18) | 0.06 |
| **IC** | -1.32 | 972.86 | .19 | 5.38 (1.03) | 5.29 (1.00) | -0.08 |
