# Supplementary material for: Psychometric evaluation of the Parental Reflective Functioning Questionnaire in Polish mothers
Source: PLoS One. 2024 Apr 17;19(4):e0299427. doi: 10.1371/journal.pone.0299427 (PMC11023587; doi:10.1371/journal.pone.0299427)
Supplement: S3 Table — Note. PM—prementalizing modes; CMS—certainty about mental states; IC—interest in and curiosity about mental states. (DOCX) [file pone.0299427.s005.docx]

|  |  |  | **Employed (*n* = 696)** | **Unemployed (*n* = 283)** |  |
| --- | --- | --- | --- | --- | --- |
|  | ***U*** | ***p*** | ***M* (*SD*)** | ***M* (*SD*)** | ***r_bp_*** |
| **PM** | 96567.00 | .63 | 2.21 (1.04) | 2.25 (1.07) | .02 |
| **CM** | 93476.50 | .21 | 4.19 (1.25) | 4.31 (1.17) | .05 |
| **IC** | 93643.50 | .23 | 5.31 (1.02) | 5.40 (1.00) | .05 |
