# Supplementary material for: Psychometric evaluation of the Parental Reflective Functioning Questionnaire in Polish mothers
Source: PLoS One. 2024 Apr 17;19(4):e0299427. doi: 10.1371/journal.pone.0299427 (PMC11023587; doi:10.1371/journal.pone.0299427)
Supplement: S4 Table — Note. PM—prementalizing modes; CMS—certainty about mental states; IC—interest in and curiosity about mental states. (DOCX) [file pone.0299427.s006.docx]

| **Variable** | ***M*** | ***SE*** | ***Mdn*** | ***SD*** | **Range** | **Minimum** | **Maximum** | **Skewness** | ***SE*** | **Kurtosis** | ***SE*** |
| --- | --- | --- | --- | --- | --- | --- | --- | --- | --- | --- | --- |
| **PM T2** | 11 | 0.05 | 2.20 | 1.16 | 6.00 | 1.00 | 7.00 | 1.06 | 0.11 | 0.90 | 0.21 |
| **CMS T2** | 4.14 | 0.05 | 4.20 | 1.22 | 6.00 | 1.00 | 7.00 | -0.19 | 0.11 | -0.42 | 0.21 |
| **IC T2** | 5.47 | 0.04 | 5.60 | 0.94 | 6.00 | 1.00 | 7.00 | -0.51 | 0.11 | 0.51 | 0.21 |
