# Supplementary material for: Psychometric evaluation of the Parental Reflective Functioning Questionnaire in Polish mothers
Source: PLoS One. 2024 Apr 17;19(4):e0299427. doi: 10.1371/journal.pone.0299427 (PMC11023587; doi:10.1371/journal.pone.0299427)
Supplement: S5 Table — Note. CMS—certainty about mental states; IC—interest in and curiosity about mental states. X—predictor; Y—dependent variable. Slope 1—regression line for lower values of a predictor; Slope 2 –regression line for higher values of a predictor. Breaking point—the value of X, which is the breaking point for the interrupted regression resulting in the two slope. (DOCX) [file pone.0299427.s007.docx]

| **X** | **Y** | **Average slope 1** | **Average slope 2** | **Breaking point** |
| --- | --- | --- | --- | --- |
| **CMS** | Attachment anxiety | β = .01, *z* = 0.21, *p* = .84 | β = -.46, *z* = -3.47, *p* < .001 | 4.60 |
|  | Attachment avoidance | β = -.17, *z* = -4.30, *p* < .001 | β = -.32, *z* = -1.81, *p =* .07 | 5.40 |
|  | Childrearing stress | β = -.22, *z* = -6.48, *p* < .001 | β = -.71, *z* = -3.28, *p* = .001 | 5.60 |
|  | Role restrictions | β = -.21, *z* = -6.18, *p* < .001 | β = -.69, *z* = -3.52, *p* < .001 | 5.60 |
|  | Borderline symptoms | β = -.03, *z* = -0.45, *p* = .65 | β = -.09, *z* = -4.20, *p* < .001 | 3.40 |
|  | Depressive symptoms | β = -.09, *z* = -3.20, *p* = .001 | β = -.28, *z* = -2.28, *p* = .02 | 5.40 |
| **IC** | Attachment anxiety | β = -.09, *z* = -1.15, *p* = .25 | β = .14, *z* = 0.84, *p* = .40 | 5.60 |
|  | Attachment avoidance | β = .02, *z* = 0.15, *p* = .88 | β = -.32, *z* = -5.89, *p* < .001 | 4.40 |
|  | Childrearing stress | β = .18, *z* = 1.47, *p* = .14 | β = -.05, *z* = -0.92, *p* = .36 | 4.60 |
|  | Role restrictions | β = .09, *z* = 0.66, *p* = .51 | β = -.19, *z* = -3.34, *p* = .001 | 4.60 |
|  | Borderline symptoms | β = -.06, *z* = -2.31, *p* = .02 | β = .12, *z* = 1.46, *p* = .15 | 6.00 |
|  | Depressive symptoms | β = .00, *z* = -0.03, *p* = .98 | β = .11, *z* = 0.86, *p* = .39 | 6.00 |
